# Amor and Psyche; a growing relationship?

**DOI:** 10.1007/s12471-012-0279-5

**Published:** 2012-04-12

**Authors:** E. E. van der Wall

**Affiliations:** 1 Interuniversity Cardiology Institute of the Netherlands (ICIN) - Netherlands Heart Institute (NHI), Utrecht, the Netherlands; 2 Department of Cardiology, Leiden University Medical Center, Albinusdreef 2 Postal zone: K5-35, P.O. Box 9600, 2300 RC Leiden, the Netherlands

Two interesting studies on the brain-heart connection were recently presented. At the 12th Annual Spring Meeting on Cardiovascular Nursing of the European Society of Cardiology (ESC) in Copenhagen, Denmark (16/17 March, 2012), Nikki Damen, a PhD student at Tilburg University, reported that patients who are depressed after coronary artery stenting have a worse prognosis. The authors reported that in patients with coronary stents depression was associated with a 1.5 times greater risk of death during a seven-year study period. The study included 1234 coronary artery disease patients in the Netherlands (mean age 62 years), who underwent assessment for depression six months after receiving a coronary stent. Depression was diagnosed in 324 (26 %) of the patients. After seven years follow-up, 187 of the patients had died, of whom 23 % of the patients with depression and 12 % of the patients without depression. The researchers found that gender and age were factors in the increased risk, with men and older patients significantly more likely to die during the study. However, the use of cholesterol-lowering statins was associated with a reduced risk of death among study participants. The intriguing Tilburg study awaits further publication.

One possible reason for the increased risk of earlier death is that depressed patients may have less healthy lifestyles in terms of smoking, drinking alcohol, exercise and diet. It could also be that depression alters the activity of the sympathetic nervous system, resulting in increased blood pressure and heart rate. Finally it was proposed that these patients may also be less likely to take their medication. The authors therefore suggested that more research is needed to determine how to screen for depression in cardiovascular patients, and then how to provide adequate treatment. However, the pertinent question here is: which treatment?

In this context, a recent paper from a Finnish group published by Honkola et al. [1] in the European Heart Journal showed that the use of psychotropic drugs, especially the combined use of antipsychotic and antidepressant drugs, was strongly associated with an increased risk of sudden cardiac death at the time of an acute coronary event. In a case–control study addressing a consecutive series of 1814 victims of sudden cardiac death (mean age 65 years), the authors compared the use of psychotropic medication between victims of sudden cardiac death and survivors of an acute coronary event. In particular it was shown that combined use of phenothiazines and any antidepressant was associated with a very high risk of sudden cardiac death (odds ratio 18.3; p < 0.001). One of the reasons may be the pro-arrhythmic effects of psychotropic drugs, resulting in prolonged QT intervals and *torsades des pointes*.

This work coming from the University of Oulu, Finland, may elucidate the observed phenomenon that psychiatric patients have an excess rate of unexplained sudden death. In an accompanying Editorial, Josep Brugada [2] wonders whether this is a population at higher risk for sudden cardiac death who should be screened and treated accordingly, or is the treatment they receive the reason for the excess mortality, and thus the drugs administered to them should be carefully selected based on the probability of a proarrhythmic effect of the therapy?

We are thus dealing with a two-edged sword. On one hand, cardiac patients with psychiatric disorders, in particular depression, are at increased risk of coronary events; on the other hand, psychotropic drugs administered to these patients can be pro-arrhythmic, especially in patients prone to myocardial ischaemia. Psychiatrists and cardiologists should work closely together to identify the optimal medication in cardiac patients with psychiatric disorders, in particular depression [3] Amor and Psyche should support each other, also in gloomy times [4, 5].
